# Specialty preference and intentions to study abroad of Syrian medical students during the crisis

**DOI:** 10.1186/s12909-018-1146-x

**Published:** 2018-03-16

**Authors:** Bisher Sawaf, Fatima Abbas, Amr Idris, Tareq Al Saadi, Nazir Ibrahim

**Affiliations:** 1 Faculty of Medicine, Syrian Private University, Mazzeh Street, P.O. Box 36822, Damascus, Syrian Arab Republic; 2 Faculty of Medicine, Damascus University, Damascus, Syria

**Keywords:** Undergraduate medical education, Postgraduate training, Specialty preferences, Syria, War, Crisis

## Abstract

**Background:**

Little research addresses how medical students develop their choice of specialty training in crisis and resource-poor settings. The newly graduated medical students determine the future of the healthcare system. This study aims to elucidate the factors influencing Syrian medical students’ specialty selection and students’ intentions to study abroad.

**Methods:**

A cross-sectional study carried out at the universities of Damascus, Al-Kalamoon and the Syrian Private University in Syria using self-administered questionnaire to investigate medical students’ specialty preferences and plans for career future. The questionnaire included questions about students’ demographic and educational characteristics, intention to train abroad, the chosen country for training.

**Results:**

Randomly selected 450 students completed the questionnaire. The two most common specialties selected were general surgery (27.6%) and internal medicine (23.5%). The most influencing factors on their decision were ‘flexibility of specialty’ (74.8%) and ‘Better work opportunities after specializing’ (69.1%). Most participants stated that they are interested in specializing abroad outside Syria (78.7%). The two most common countries of choice were Germany (35.5%) and the United States of America (24.6%). Acquiring a visa to the foreign country was the most common obstacle of specializing abroad (*n* = 186, 53.6%). Male gender, having a previous clinical training abroad, and having friends or relatives living abroad were significant factors in predicting students’ interest in specializing abroad.

**Conclusion:**

Internal medicine and surgery are the most reported specialties of choice in this study and most of the participants reported intentions to study abroad. Their specialty preferences are influenced by both familiar epidemiological and war-driven factors. These data can be useful to design further cohort study to understand the war-related affecting factors on students’ plans for their career in the effort of improving the balance of healthcare system in Syria.

**Electronic supplementary material:**

The online version of this article (10.1186/s12909-018-1146-x) contains supplementary material, which is available to authorized users.

## Background

Global orientation is now towards specialization [1]. In poor-resource countries this may affect primary care which is a phenomenon maybe caused by the little exposure to primary care in medical school [2]. Published data about students’ preferences revealed that surgery, internal medicine, obstetrics and gynecology (OB/GYN) and pediatrics are the most commonly reported choices by medical students [3–7]. Decision in choosing a specialty is commonly influenced by both personal characteristics and academic culture and experiences (including students’ interaction with friends, patients, attending physicians and academic professors) [8, 9]. Numerous research concentrated on cultural and social values and specific groups such as women in making the career choice [10–12] Many studies revealed that females have a higher tendency to select specific specialties such as OB/GYN, pediatrics, anesthesia and radiology, where they find suitable lifestyle that allows them to do their familial duties [3, 4, 7, 13–17]. On the other hand, males tend to choose surgery, internal medicine and orthopedics for the necessity for high paying jobs and proper lifestyle [3, 4, 7, 13–17].

As described in studies conducted in Turkey [7], Jordan [3], Saudi Arabia [18], Taiwan [19], Pakistan [20] and India [21], lifestyle is one of the factors greatly influencing students’ preferences. Prestige and financials were the most important factors for Turkish students [7], while personal interest in the specialty was the essential factor for students in Saudi Arabia [18], Taiwan [19], Pakistan [20] and India [21]. Lifestyle may also incorporate factors like personal time free from practice, requirements for leisure, family and avocational pursuits, remuneration and length of training [17].

Medical education in the Syrian universities consists of a 6-year educational program. The first 3 years cover the basic-sciences-related subjects, while in the 4th, 5th years students undergo clinical clerkships in the university hospitals. These clerkships are repeated in the 6th year having 2 months of training in each main specialty [22]. Then, Syrian medical students are required to pass a standardized medical exam for graduation from Syrian medical schools at the end of the sixth year [22]. Subsequently, they will are allocated to different residency programs according to their scores in this exam in addition to their medical school cumulative grades in the context of their desires.

With the advances in delivering the healthcare worldwide, studying career preferences is important to aid planning the educational programs and setting the plan for balanced healthcare workforce that is significantly impaired in the times of crisis such like Syria’s. Physicians’ human rights estimated that 50% of the physicians have left the country, while hundreds have been killed or injured [23]. And although migration of medical workers mainly from developing countries in which the available resources for both health workers and users are limited to more developed ones is a worldwide problematic phenomenon [24–26], in crisis the need for human resources is inevitable [5, 27]. More population are expected to need primary and health care that is lacking to deliver especially in the remote sites [2, 28].

In Greece during the economic crisis, reports revealed a high percentage of medical students planning to specialize in other countries like Germany causing depletion and shortages in the workforce [2, 27]. Training abroad is the major factor to drain medical graduates’ intentions for migration. This has been studied with special interest but less reported in times of conflicts or countries during the wars [25, 29].

The damage in the Syrian health system infrastructure, the overwhelmed system, poor job market, financial concerns and losing huge numbers of educators, physicians and medical workers [30, 31] resulted in an unequal distribution of the healthcare personnel capacity into all the specialties and areas where healthcare is needed. This study investigates the Syrian medical students’ attitudes, factors influencing their specialty choices and their plans about leaving Syria to specialize abroad in order to help policy makers in Syria to plan for a well-designed educational orientation and better healthcare system.

## Methods

### Study design and participants

A cross-sectional study was conducted in August 2016 at the Faculty of Medicine of the three universities in Damascus, Syria. The universities involved were Damascus University, Syrian Private University, and University of Al-Kalamoon.

All participants were current medical students. Participants were recruited from the fourth- through sixth-year of study using a convenience sampling method. First- through third-year students were not included in the study because we thought that they would not be having clear plans about post-graduate training.

Medical students were approached during their medical training sessions and were asked to participate in the study. Participants were informed about the objectives of the study and that participation was voluntary and anonymous. They were asked to provide written consent to participate in the study prior to completing the questionnaire.

### The questionnaire

All questions were administered to students in Arabic language as Arabic is the native language of the Syrian medical students. The first part of the questionnaire contained questions about the demographic characteristics of the participants.

The second part of the questionnaire contained questions about the students’ postgraduate plans regarding choosing their future specialty, training location, their plans after finishing their medical training and all the contributing factors in their decision. Possible influences were selected on the basis of literature reviews and discussions with medical students and physicians.

Overall, the questionnaire contained 49 questions. Not all questions were required to be answered since they were not applicable to some participants who were not considering completing their post-graduate training abroad. We excluded the questionnaires that were returned completely empty. Due to having some missing values, the number and percentage of the missing responses or the total number of participants who completed each question were added to the results.

### Statistical analysis

Two authors entered data into Microsoft Excel sheet using a Google online survey form. Data were then imported into the Statistical Package for Social Sciences version 22.0 (SPSS Inc., Chicago, IL, United States) for analysis.

Data were reported as frequencies and percentages (for categorical variables) or means and standard deviations (for continuous variables). For questions with the option of choosing multiple responses, we reported the percentage of cases (participants who answered each corresponding question).

A binary logistic regression analysis was conducted to predict participants’ probabilities of being interested to complete their post-graduated training abroad based on their characteristics. The dependent variable was the presence of interest to complete their medical training abroad, and the independent variables including the investigated characteristics of the participants. The results of logistic regression were reported as odds ratio (ORs) with 95% confidence intervals (CI).

## Results

Overall, 450 students completed the questionnaire and agreed to participate in the study with a response rate of 54%. Four responses were completely empty and were discarded. We included 446 responses in the final analysis.

Participants’ characteristics are summarized in Appendix. Students were from fourth (83 students; 18.6%), fifth (176 students; 39.5%), and sixth (181 students; 40.6%) year of study in medical school. Mean age (± SD) of the participants was 23.44 ± 1.4 years.

More than half of the participants ranked themselves academically among the middle third of students based on their medical school grades (251 students; 56.3%).Only a small percentage reported that they had clinical training abroad (31 students; 7.0%) and more than 40% of the students stated that they are planning to do so.

### Type of specialty

**Fig. 1:**
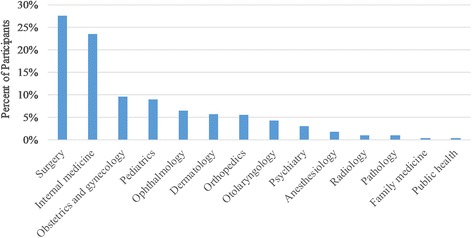
Students’ specialty of choice after graduation

The two most common chosen specialties by students were general surgery (27.6%) and internal medicine (23.5%) (Fig. 1). The factors in choosing the students’ desired specialty are summarized in Appendix.

Factors that were most commonly agreed or strongly agreed on their importance in their decision were ‘flexibility of work schedule’ (74.8%), ‘easiness of finding jobs after specializing’ (69.1%), ‘anticipated income/salary’ (67.5%), ‘training hours’ (65%), ‘reputation of the specialty’ (63%), ‘interaction with patients’ (62.3%), and ‘diversity of patients’ (60.3%). On the other hand, factors that were most commonly disagreed or strongly disagreed on their importance in their decision were ‘workload’ (22.5%), ‘interest in research’ (15.9%), and ‘length and difficulty of the residency program’ (15.5%).

Students’ choice was significantly affected by their gender. Pathology, dermatology, pediatrics, and OB/GYN were mainly chosen as the specialty of choice by females. All who went for pathology as their specialty of choice were females. Among those who have chosen dermatology, 80.8% were females, 75.0% for pediatrics, 74.4% for OB/GYN, 61.5% for psychiatry and 57.1% for anesthesia. On the other hand, public health, orthopedic surgery, general surgery, and radiology were mainly chosen by males. (100% of those who have chosen public health were males, 96% for orthopedics, and 88.4% for surgery).

### Post-graduate training abroad

**Fig. 2:**
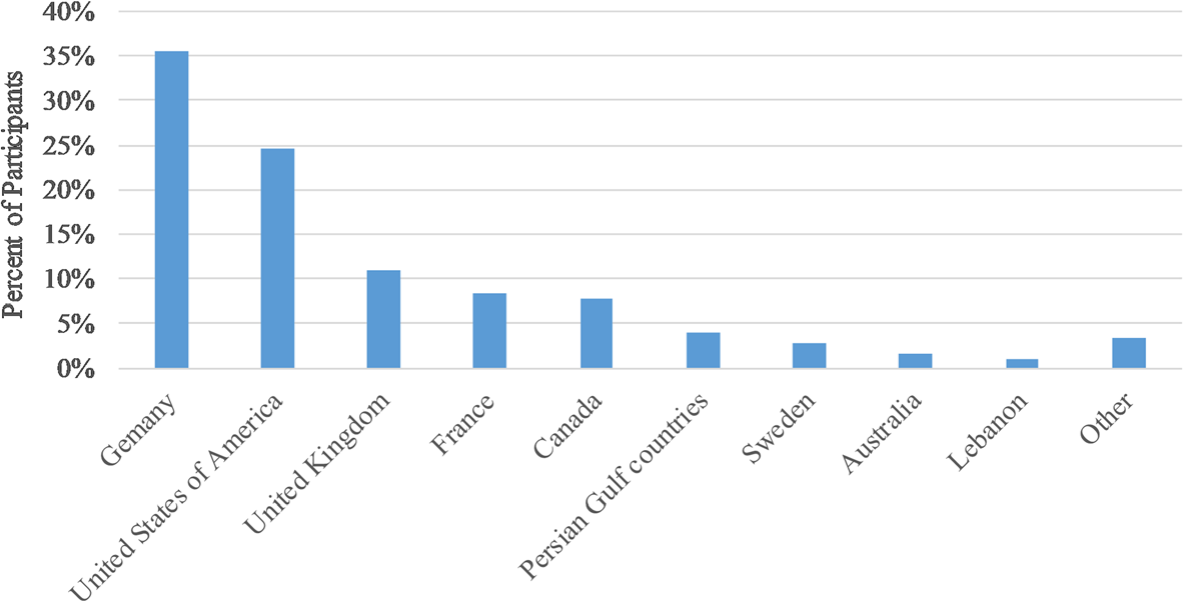
Students’ country of choice for specializing abroad^†^. †: only those who were interested in specializing abroad answered the question

Participants’ characteristics and plans regarding completing their training abroad are summarized in Table 1. Most participants stated that they are interested in training abroad (78.7%). The two most common countries chosen were Germany (35.5%) and the United States of America (24.6%) (Fig. 2). The most common important factors in deciding to study abroad were ‘having a better medical training’ (40.2%) and ‘having a secured job’ (32.6%). On the other hand, these students varied widely in their plans after completing their training abroad (Table 1), with most of them planning to work either less than 5 years (24.7%) or between 5 and 10 years (21.6%) before returning to Syria, or to never return to Syria (22.2%).

**Table 1:** Participants’ characteristics and plans regarding specializing abroad

	Number	Percent
Interested in specializing abroad		
Yes	351	78.7%
No	92	20.6%
Missing	3	0.7%
Plans after specializing abroad^b^		
Returning to home country directly after specialization.	68	19.1%
Working less than 5 years abroad then returning to home country.	88	24.7%
Working less than 5–10 years abroad then returning to home country.	77	21.6%
Working more than 10 years abroad then returning to home country.	44	12.4%
Never return to home country.	79	22.2%
The most important goal of specializing abroad^b^		
Having a better medical training	143	40.2%
Having a secured job	116	32.6%
Acquiring another nationality	29	8.1%
Having higher income	36	10.1%
Ability to work in another country	32	9.0%
Important obstacles of specializing abroad^a^		
Costs of travelling.	122	35.2%
Required exams in foreign country before specializing.	125	36.0%
Acquiring a visa to the foreign country.	186	53.6%
Adapting with new communities.	95	27.4%
Finding good specializing opportunities.	75	21.6%
Do you have any relatives or friends who are living abroad and would help you specializing in the country they are living in?		
Yes	258	57.8%
No	185	41.5%
Missing	3	0.7%
Are you encouraged by university professors to specialize abroad?		
Yes	166	37.2%
No	275	61.7%
Missing	5	1.1%
Do you have university professors who would help you specializing abroad?		
Yes	68	15.2%
No	375	84.1%
Missing	3	0.7%
Does watching your colleagues specializing abroad encourage you to do so?		
Yes	286	64.1%
No	151	33.9%
Missing	9	2.0%
About the language of the country in which you plan to specialize		
I wish to specialize in an Arabic-speaking country.	59	13.2%
I wish to specialize in a non-Arabic speaking country, and I have not learnt its language yet.	245	54.9%
I wish to specialize in a non-Arabic speaking country, and I am learning its language.	89	20.0%
I wish to specialize in a non-Arabic speaking country, and I can speak its language fluently.	39	8.7%
Missing	14	3.1%
Source of information about specializing abroad^a^		
Media (movies, series, websites…).	134	33.3%
Relatives or friends living abroad.	209	52.0%
My personal observations while having clinical training abroad.	20	5.0%
Students who had clinical training abroad.	128	31.8%
Doctors who are specialized/currently specializing abroad.	155	38.6%
By comparing between those who specialized in Syria with those who specialized abroad.	129	32.1%

*N* number of participants who chose the corresponding answer

%: percent of participants who chose the corresponding answer

*SD* Standard Deviation

^a^choosing more than one option was allowed

^b^only those who were interested in specializing abroad answered the question

Acquiring a visa to the country of desire was the most common obstacle to complete the post-graduate training abroad (53.6%).

When investigating the role of students’ relatives or friends who live in the country of choice for specializing abroad, we found that most students have such people that would help them specializing in the country they are living in (57.8%), and most of them chose such people as the source of information about medical training abroad (52.0%).

However, most of the students reported that they do not have a professors or an attending physician who would help them or provide enough information to complete their medical training abroad (84.1%) and felt that they were not encouraged by their university professors to complete their training abroad (61.7%). On the other hand, most students were encouraged to specialize abroad by watching their colleagues doing that (64.1%).

Students’ answers about the importance of different factors in encouraging them to choose specializing abroad are summarized in Appendix. Factors that were most commonly agreed or strongly agreed on their importance were ‘training opportunities’ (73.5%), ‘clinical training quality and content’ (73.5%), ‘working conditions after residency’ (69.2%), ‘work opportunities’ (69%), ‘learning in the specialty programs’ (68.4%), ‘effect of residency training on working after residency’ (68%), and ‘financial status for doctors after residency’ (67.9%). On the other hand, factors that were most commonly disagreed or strongly disagreed on their importance were ‘social issues related to social customs and traditions’ (13.2%), ‘research training’ (11.2%), ‘financial status for residents’ (11%), and ‘personal issues related to partner, parents, children’ (11%).

Table 2 shows the model of binary logistic regression for the interest in specializing abroad. Gender, clinical training abroad, and the help of abroad friends or relatives were the statistically significant factors in predicting students’ interest in specializing abroad. Males compared to females were significantly about 2 times more likely to be interested in specializing abroad (OR = 1.893; 95% CI 1.049–3.419). In addition, those who stated that they have not had any clinical training abroad yet, but they are planning to do so, were significantly about 9 times more likely to be interested in specializing abroad compared to those with no previous clinical training abroad and no intention to do so (OR = 9.273; 95% CI 4.291–20.040). However, this significance was not present for those who reported having had clinical training abroad before (OR = 3.058; 95% CI 0.806–11.599). Furthermore, those who have relatives or friends who are living abroad and would help them specializing abroad were significantly about two times more likely to be interested in specializing abroad than those who have not (OR = 2.617; 95% CI 1.427–4.799).

**Table 2:** Factors associated with participants’ interest in specializing abroad

	*P*-value	AOR	95% C.I. for AOR	
			Lower	Upper
Gender				
Female (ref.)				
Male	0.034^*^	1.893	1.049	3.419
Current university	0.955			
Damascus University (ref.)				
Syrian Private University	0.762	0.896	0.441	1.821
University of Kalamoon	0.905	0.952	0.425	2.132
Year of study	0.240			
Fourth (ref.)				
Fifth	0.311	1.547	0.664	3.603
Sixth	0.782	0.892	0.399	1.997
Ranking estimation based on medical school grades	0.417			
In the lower third of students (ref.)				
In the middle third of students	0.187	0.562	0.239	1.322
In the higher third of students	0.367	0.640	0.242	1.689
Nationality	0.449			
Syrian nationality (ref.)				
Both Syrian and non-Syrian nationality	0.926	1.048	0.388	2.834
Non-Syrian nationality	0.206	2.801	0.568	13.805
Financial status	0.421			
Bad (ref.)				
Below average	0.682	0.659	0.089	4.856
Average	0.797	1.286	0.190	8.710
Good	0.520	1.914	0.264	13.855
Excellent	0.702	1.502	0.187	12.064
Residence				
Countryside (ref.)				
City	0.870	1.056	0.549	2.032
Marital status	0.106			
Single (ref.)				
In a relationship	0.323	1.460	0.689	3.097
Married	0.075	0.268	0.063	1.144
Had clinical training abroad	> 0.001^*^			
No, and not planning to do so (ref.)				
No, but planning to do so	> 0.001^*^	9.273	4.291	20.040
Yes	0.100	3.058	0.806	11.599
Have relatives or friends who are living abroad and would help specializing abroad				
No (ref.)				
Yes	0.002^*^	2.617	1.427	4.799
Encouraged by university professors to specialize abroad				
No (ref.)				
Yes	0.168	1.609	0.819	3.161
Have university professors who would help specializing abroad				
No (ref.)				
Yes	0.495	0.746	0.321	1.732
Constant	0.767	0.726		

Abbreviations:

*AOR* Adjusted odds ratio

*CI* Confidence interval

*Ref* reference group

*. Significant at the 0.05 level

## Discussion

To our knowledge, this is the first study to investigate the specialty preferences and plans of Syrian medical students for their career. There are no pre-crisis data to compare with but this study may be important for future research in this area.

Surgery, internal medicine, OB/GYN, orthopedics and pediatrics were the most common choices selected by the students (approximately 80%). These findings were similar to the published studies in other countries such as Jordan [3], Sudan [32] and Pakistan [4]. Surgery is still one of the most reported specialties of choice despite the fact that it has very difficult lifestyle and huge workload. This may return to the influence of role models, prestige and the high anticipated income that it attracts the majority of students.

The majority of students in our study were affected by the flexibility of work schedule, easiness of finding jobs after specializing, training hours and reputation, and this also was reflected in many published studies [16, 32]. While reputation, prestige, lifestyle, and financial aspects were considered important reasons in our study, they were not the only main factors; specialty opportunities and financial concerns were also important for a large number of the students (Additional file 1).

Some specialties like psychiatry and public health are poorly reported by students as a preference despite the huge need for such specialties in poor-resource countries especially in crisis. The poor orientation toward these specialties maybe the reason created this attitude. To supply the needs in primary healthcare, shift in education towards primary care is required. On the other hand, we found that pathology, dermatology, pediatrics, and OB/GYN were mainly chosen as the specialty of choice by females, while surgery, orthopedics, public health, and radiology were mainly chosen by males. Gender distribution difference is well investigated in other studies with similar findings explained with the tendency of females to select specialties which provide them with comfortable life style while males tend to go for highly paying jobs [1].

Although we did not follow up students, it is reported that medical students’ first preference differs as they progress through the years of study to be less based on social perception and to be fairly distributed in the different specialties [3]. This is possibly due to the effect of introducing students to different specialties upon advancing in their medical training and the impact that has been made by the residents or the physicians at each training program as also suggested by other studies [33, 34].

Having well-trained doctors is a critical need in times of war in order to fulfill the requirements of the burdened healthcare system. However, it seems that most students (78.8%) are planning for specializing abroad. Although there is no data to identify the increased percentage of students planning to travel for studying from the pre-crisis years, number of practicing physicians is diminished comparable with the pre-crisis situations [23]. Despite that the rate of migration intentions is consistent with data from other countries, it is a little higher in our study assumingly because of the crisis impact [35, 36].

Factors affecting migration intentions might include those discussed in other similar studies [2–4, 27, 32] such as lifestyle, personal interest and income in the host country in addition to the current crisis-related circumstances such as feeling of fear and insecurity, and the possibility of being displaced or injured. War-driven economic deterioration is another potential factor contributing to such choice; increased living cost, along with the very low income of the Syrian residents -regardless of their specialties- are key financial issues possibly encouraging physicians’ immigration [31].

Also, medical training in Syria now is limited to be located in the safe and functional areas, mostly in Damascus. With the increase rate of medical graduates, oversaturation in the job market and the residency opportunities, salaries, quality of training may attribute to push students toward studying abroad. Students’ best preferences of specialty might also be restricted regarding their scores in the national exam and the college cumulative average. This might also enhance them to opt for studying abroad plans in order to obtain medical training in the desired specialty.

On the other hand, most of the students who intend to study abroad reported their desire to return to Syria with very different timetable plans. As having this desire, their intention to study abroad can be explained with their eagerness to have better training and opportunities within the current circumstances. Few students reported the desire to not return, which might be because of personal or academic plans.

The two most common countries of choice for specializing were Germany (35.5%) and the United States (24.6%) (Fig. 2). That was similar to the results of many other studies [27], possibly because of the high payment, the huge capacity and the advanced training in those countries which attract those with great passion and high skills in what they do [2]. Students reported that they are welling to learn the language of the destination country since the most of students are intending to study in non-Arabic country. Earlier language learning or cultural preferences might also affect this choice of destination country. Nevertheless, our students reported many difficulties facing them in specializing abroad. The major problem was getting a visa to enter these countries. Medical students have to apply many times before they get approved for their visas. Recently, an executive order by the United States of America (U.S.A) president was released to ban some nationalities from entering USA and this involved Syrians. As a result, hundreds of medical students are negatively affected and endangered of losing their chance to complete their study in USA as they are aspiring [6].

We found that students’ desire to study abroad is influenced by many factors. Males were more likely that females to seek specializing abroad possibly due to the Syrian cultural habits which place more life responsibilities on males and provide more with less restrictions on travelling compared to females, also because of security issues in a country of conflicts,. Therefore, males are found to be forced to study abroad for the sake of better and secure life and better medical training. Another significant factor was having clinical training abroad before graduation which potentially increases students’ connections and ambition to continue their post-graduate studying in other countries. Similarly, having friends and relatives in other countries was also a significant factor, possibly due to their role in supporting students in many ways especially in financial issues, living-abroad advices and training experience abroad. Negative role of faculty’s staff members was reported by students in our study to intensify their intentions to study abroad.

### Limitation

We did not investigated crisis related factors affecting students’ selection preferences in or out of Syria since we aimed to chart general spectrum of their plans for career. Focused research should be done to study these factors and search for solutions for them.

One limitation for this study is the cross-sectional design that cannot capture changes in career preferences over time. A prospective cohort study following these students through medical school and assessing their career preferences might yield more information about the type and place of specialty that students ended up with. Furthermore, there is no prior survey before crisis to compare our results with. However, our findings were consistent with published data from low income countries, and countries suffering from economic crisis like Greece. In addition, the convenience sampling method and the relatively small sample size might limit the generalizability of the results.

## Conclusions

In general, factors affecting Syrian medical students’ choices about specialization were comparable to those reported for regional countries. However, this study is alarming in the terms of having imbalanced healthcare system in Syria due to students’ preferences and their intentions to study abroad which increases the gap between the demand and supply of physicians. Policy makers should plan for better distribution strategies such as residency capacity, quality, and higher income for residents, better working circumstances and educational orientation for the sake of re-building the healthcare system in Syria.

## Additional file

Additional file 1: Participants’ characteristics and opinions about the importance of different factors in choosing the post-graduation specialty and in encouraging them to choose specializing abroad. (DOCX 23 kb)

## Abbreviations

CI: Confidence Intervals
OB/GYN: Obstetrics and Gynecology
ORs: Odds Ratio
U.S.A: United States of America

## References

[1] Azu, O.O., E. Naidu, and J. Naidu, Choice of speciality amongst first-year medical students in the Nelson R. Mandela School of Medicine*,* University of KwaZulu-Natal 2013. Vol. 5. 2013.

[2] Ifanti AA (2014). Physicians’ brain drain in Greece: a perspective on the reasons why and how to address it. Health Policy.

[3] Khader Y (2008). Factors affecting medical students in formulating their specialty preferences in Jordan. BMC Med Educ.

[4] Rehman A (2011). *Pakistani medical students' specialty preference and the influencing factors.* JPMA. J Pak Med Assoc.

[5] Akl EA (2008). Post-graduation migration intentions of students of Lebanese medical schools: a survey study. BMC Public Health.

[6] Fouad FM, et al. Health workers and the weaponisation of health care in Syria: a preliminary inquiry for the lancet-American University of Beirut Commission on Syria. Lancet. 2017;390:2516–2. 10.1016/S0140-6736(17)30741-9 28314568

[7] Fevzi Dikici M (2008). Factors affecting choice of specialty among first-year medical students of four universities in different regions of Turkey. Croatian Med J.

[8] Saigal P (2007). Factors considered by medical students when formulating their specialty preferences in Japan: findings from a qualitative study. BMC Med Educ.

[9] Knox KE (2008). Short report: factors that affect specialty choice and career plans of Wisconsin's medical students. WMJ.

[10] Gorenflo DW, Ruffin MT, Sheets KJ (1994). A multivariate model for specialty preference by medical students. J Fam Pract.

[11] Harris MG, Gavel PH, Young JR (2005). Factors influencing the choice of specialty of Australian medical graduates. Med J Aust.

[12] Dorsey ER, Jarjoura D, Rutecki GW (2005). The influence of controllable lifestyle and sex on the specialty choices of graduating U.S. medical students, 1996-2003. Acad Med.

[13] Alers M (2014). Speciality preferences in Dutch medical students influenced by their anticipation on family responsibilities. Perspectives Med Educ.

[14] Alawad AAMA, et al. Factors considered by undergraduate medical students when selecting specialty of their future careers. Pan Afr Med J. 2015;20:102. 10.11604/pamj.2015.20.102.4715 PMC4458322 26090050

[15] Baxter N, Cohen R, McLeod R. The impact of gender on the choice of surgery as a career. Am J Surg. 1996;172(4):373–6. 10.1016/S0002-9610(96)00185-7 8873533

[16] Mehmood SI (2012). Specialty preferences: trends and perceptions among Saudi undergraduate medical students. Med Teach.

[17] Mwachaka P.M, and E.T. Mbugua. Specialty preferences among medical students in a Kenyan university. Pan Afr Med J. 2010;5(1):18. PMC3032620 21293745

[18] Abdulghani HM (2013). What determines the selection of undergraduate medical students to the specialty of their future careers?. Med Teach.

[19] Chang PY (2006). Factors influencing medical students' choice of specialty. J Formos Med Assoc.

[20] Huda N, Yousuf S (2006). Career preference of final year medical students of Ziauddin medical university. Educ Health (Abingdon).

[21] Subba S (2012). Future specialization interests among medical students in southern India.

[22] Idris A (2016). Self-reported study habits for enhancing medical students’ performance in the National Medical Unified Examination. Avicenna J Med.

[23] Rights, P.f.H. Less Than a Third of Aleppo’s Hospitals Functioning; 95% of Doctors Have Fled, Been Detained or Killed. 2015 [cited 2017 05 July]; Available from: http://physiciansforhumanrights.org/press/press-releases/less-than-a-third-of-aleppos-hospitals-functioning-95-of-doctors-have-fled-been-detained-or-killed.html.

[24] Gupta N (2003). Assessing human resources for health: what can be learned from labour force surveys?. Hum Resour Health.

[25] Mullan F (2005). The metrics of the physician brain drain. N Engl J Med.

[26] Akl EA (2007). The United States physician workforce and international medical graduates: trends and characteristics. J Gen Intern Med.

[27] Labiris G (2014). Perceptions of Greek medical students regarding medical profession and the specialty selection process during the economic crisis years. Health Policy.

[28] Puertas EB, Arosquipa C, Gutierrez D (2013). Factors that influence a career choice in primary care among medical students from high-, middle-, and low-income countries: a systematic review. Rev Panam Salud Publica.

[29] Sheikh A (2012). Physician migration at its roots: a study on the factors contributing towards a career choice abroad among students at a medical school in Pakistan. Glob Health.

[30] Kherallah M (2012). Health care in Syria before and during the crisis. Avicenna J Med.

[31] Organization, W.H. WHO Annual report 2016 on health humanitarian response in the Syrian Arab Repbulic. Humanitarian Health Action: Syrian Arab Republic situation reports 2016 [cited 2017 05 July]; Available from: http://www.who.int/hac/crises/syr/sitreps/syria_annual-report-2016.pdf?ua=1.

[32] Alawad AA (2015). Factors considered by undergraduate medical students when selecting specialty of their future careers. Pan Afr Med J.

[33] McManus C (1997). Medical careers: stories of a life. Med Educ.

[34] Zeldow P, Preston R, Daugherty S (1992). The decision to enter a medical specialty: timing and stability. Med Educ.

[35] Syed NA (2008). Reasons for migration among medical students from Karachi. Med Educ.

[36] Imran N (2011). Brain drain: post graduation migration intentions and the influencing factors among medical graduates from Lahore, Pakistan. BMC Res Notes.

