# Supplementary material for: Specialty preference and intentions to study abroad of Syrian medical students during the crisis
Source: BMC Med Educ. 2018 Mar 16;18:39. doi: 10.1186/s12909-018-1146-x (PMC5857144; doi:10.1186/s12909-018-1146-x)
Supplement: Supplementary file 1 — Participants’ characteristics and opinions about the importance of different factors in choosing the post-graduation specialty and in encouraging them to choose specializing abroad. (DOCX 23 kb) [file 12909_2018_1146_MOESM1_ESM.docx]

Appendix File:

Table 1. Participants’ characteristics.

| Characteristic | N or Mean | % or ± SD |
| --- | --- | --- |
| Age (missing: 9) | 23.44 | ±1.4 |
| Gender | | |
| Female | 175 | 39.2% |
| Male | 266 | 59.6% |
| Missing | 5 | 1.1% |
| Current university | | |
| University of Damascus | 247 | 55.4% |
| Syrian Private University | 110 | 24.7% |
| University of Kalamoon | 86 | 19.3% |
| Missing | 3 | 0.7% |
| Year of study | | |
| Fourth | 83 | 18.6% |
| Fifth | 176 | 39.5% |
| Sixth | 181 | 40.6% |
| Missing | 6 | 1.3% |
| Ranking estimation based on medical school grades | | |
| In the lower third of students | 70 | 15.7% |
| In the middle third of students | 251 | 56.3% |
| In the higher third of students | 120 | 26.9% |
| Missing | 5 | 1.1% |
| Nationality | | |
| Syrian nationality | 375 | 84.1% |
| Non-Syrian nationality | 18 | 4.0% |
| Both Syrian and non-Syrian nationality | 47 | 10.5% |
| Missing | 6 | 1.3% |
| Financial status | | |
| Bad | 11 | 2.5% |
| Below average | 37 | 8.3% |
| Average | 222 | 49.8% |
| Good | 100 | 22.4% |
| Excellent | 74 | 16.6% |
| Missing | 2 | 0.4% |
| Residence | | |
| City | 320 | 71.7% |
| Countryside | 112 | 25.1% |
| Missing | 14 | 3.1% |
| Marital status | | |
| Single | 346 | 77.6% |
| In a relationship | 84 | 18.8% |
| Married | 13 | 2.9% |
| Missing | 3 | 0.7% |
| Did you have clinical training abroad? | | |
| Yes | 31 | 7.0% |
| No, but I am planning to do so. | 188 | 42.2% |
| No, and I am not planning to do so. | 223 | 50.0% |
| Missing | 4 | 0.9% |
| N: number of participants who chose the corresponding answer.  %: percent of participants who chose the corresponding answer.  SD: Standard Deviation. | | |

Table 2. Students’ opinions about the importance of different factors in choosing the post-graduation specialty.

|  | Strongly agree | | Agree | | Neutral | | Disagree | | | Strongly disagree | | Missing | |
| --- | --- | --- | --- | --- | --- | --- | --- | --- | --- | --- | --- | --- | --- |
|  | N | % | N | % | N | % | | N | % | N | % | N | % |
| Training hours. | 154 | 34.5% | 136 | 30.5% | 95 | 21.3% | | 33 | 7.4% | 12 | 2.7% | 16 | 3.6% |
| Flexibility of work schedule. | 130 | 29.1% | 204 | 45.7% | 73 | 16.4% | | 20 | 4.5% | 4 | 0.9% | 15 | 3.4% |
| Reputation of the specialty. | 136 | 30.5% | 145 | 32.5% | 113 | 25.3% | | 29 | 6.5% | 11 | 2.5% | 12 | 2.7% |
| Curriculum of residency program. | 90 | 20.2% | 177 | 39.7% | 111 | 24.9% | | 36 | 8.1% | 8 | 1.8% | 24 | 5.4% |
| Length and difficulty of residency program. | 84 | 18.8% | 122 | 27.4% | 151 | 33.9% | | 57 | 12.8% | 12 | 2.7% | 20 | 4.5% |
| Workload. | 80 | 17.9% | 121 | 27.1% | 128 | 28.7% | | 81 | 18.2% | 19 | 4.3% | 17 | 3.8% |
| Interest in research. | 107 | 24.0% | 141 | 31.6% | 106 | 23.8% | | 55 | 12.3% | 16 | 3.6% | 21 | 4.7% |
| Easiness of finding jobs after specializing. | 173 | 38.8% | 135 | 30.3% | 85 | 19.1% | | 28 | 6.3% | 6 | 1.3% | 19 | 4.3% |
| Interest in long-term relationship with patients. | 106 | 23.8% | 143 | 32.1% | 120 | 26.9% | | 47 | 10.5% | 5 | 1.1% | 25 | 5.6% |
| Interaction with patients. | 133 | 29.8% | 145 | 32.5% | 103 | 23.1% | | 36 | 8.1% | 7 | 1.6% | 22 | 4.9% |
| Diversity of patients. | 111 | 24.9% | 158 | 35.4% | 112 | 25.1% | | 32 | 7.2% | 7 | 1.6% | 26 | 5.8% |
| Anticipated income/salary. | 152 | 34.1% | 149 | 33.4% | 88 | 19.7% | | 17 | 3.8% | 21 | 4.7% | 19 | 4.3% |
| Presence of role models (family/teachers). | 132 | 29.6% | 128 | 28.7% | 106 | 23.8% | | 38 | 8.5% | 21 | 4.7% | 21 | 4.7% |
| Advice of professors, colleagues or family. | 108 | 24.2% | 131 | 29.4% | 125 | 28.0% | | 38 | 8.5% | 22 | 4.9% | 22 | 4.9% |
| N: number of participants who chose the corresponding answer.  %: percent of participants who chose the corresponding answer. | | | | | | | | | | | | | |

Table 3. Students’ opinions about the role of different factors in encouraging them to choose specializing abroad.

|  | Strongly agree | | Agree | | Neutral | | Disagree | | | Strongly disagree | | Missing | |
| --- | --- | --- | --- | --- | --- | --- | --- | --- | --- | --- | --- | --- | --- |
|  | N | % | N | % | N | % | | N | % | N | % | N | % |
| **Factors related to medical training as a resident:** | | | | | | | | | | | | | |
| Training opportunities. | 175 | 39.2% | 153 | 34.3% | 77 | 17.3% | | 22 | 4.9% | 6 | 1.3% | 13 | 2.9% |
| Clinical training quality and content. | 150 | 33.6% | 178 | 39.9% | 79 | 17.7% | | 25 | 5.6% | 3 | 0.7% | 11 | 2.5% |
| Research training opportunities. | 121 | 27.1% | 151 | 33.9% | 109 | 24.4% | | 35 | 7.8% | 15 | 3.4% | 15 | 3.4% |
| Learning in the specialty programs. | 128 | 28.7% | 177 | 39.7% | 95 | 21.3% | | 33 | 7.4% | 3 | 0.7% | 10 | 2.2% |
| Working conditions as a resident. | 134 | 30.0% | 155 | 34.8% | 118 | 26.5% | | 23 | 5.2% | 6 | 1.3% | 10 | 2.2% |
| Financial status for residents. | 158 | 35.4% | 125 | 28.0% | 104 | 23.3% | | 42 | 9.4% | 7 | 1.6% | 10 | 2.2% |
| Effect of residency training on working after residency. | 164 | 36.8% | 139 | 31.2% | 97 | 21.7% | | 25 | 5.6% | 11 | 2.5% | 10 | 2.2% |
| **Factors related to working after residency:** | | | | | | | | | | | | | |
| Work opportunities. | 163 | 36.5% | 145 | 32.5% | 114 | 25.6% | | 21 | 4.7% | 3 | 0.7% | 0 | 0% |
| Working conditions after residency. | 142 | 31.8% | 167 | 37.4% | 107 | 24.0% | | 26 | 5.8% | 4 | 0.9% | 0 | 0% |
| Financial status for doctors after residency. | 174 | 39.0% | 129 | 28.9% | 114 | 25.6% | | 22 | 4.9% | 7 | 1.6% | 0 | 0% |
| **Other factors:** | | | | | | | | | | | | | |
| Personal issues (related to partner, parents, children). | 105 | 23.5% | 131 | 29.4% | 144 | 32.3% | | 38 | 8.5% | 11 | 2.5% | 17 | 3.8% |
| Social issues (related to social customs and traditions). | 82 | 18.4% | 143 | 32.1% | 147 | 33.0% | | 41 | 9.2% | 18 | 4.0% | 15 | 3.4% |
| Safety issues (related to the Syrian crisis). | 135 | 30.3% | 120 | 26.9% | 140 | 31.4% | | 19 | 4.3% | 15 | 3.4% | 17 | 3.8% |
| N: number of participants who chose the corresponding answer.  %: percent of participants who chose the corresponding answer. | | | | | | | | | | | | | |
